# Glucagon-like peptide-1 receptor agonists are associated with cardiac, cancer- and mortality-related benefits in diabetic patients treated with anthracyclines

**DOI:** 10.1093/ehjopen/oeag109

**Published:** 2026-06-25

**Authors:** Sunnia T Chen, Teodora Donisan, Bradley R Lewis, Vidur Kailash, Sandra Herrmann, Kathryn J Ruddy, David Yat-Chung Cheung, Danielle Desautels, Davinder S Jassal, Joerg Herrmann

**Affiliations:** Department of Internal Medicine, Mayo Clinic, 200 First Street SW, Rochester, MN 55905, USA; Department of Cardiovascular Medicine, Mayo Clinic, 200 First Street SW, Rochester, MN 55905, USA; Division of Clinical Trials and Biostatistics, Mayo Clinic, 200 First Street SW, Rochester, MN 55905, USA; College of Osteopathic Medicine, Touro University, 1310 Club Drive, Vallejo, CA 94592, USA; Department of Internal Medicine, Mayo Clinic, 200 First Street SW, Rochester, MN 55905, USA; Department of Oncology, Mayo Clinic, 200 First Street SW, Rochester, MN 55905, USA; Department of Physiology and Pathophysiology, University of Manitoba, 745 Bannatyne Avenue, Winnipeg, MB, Canada R3E 0J9; Paul Albrechtsen Research Institute, CancerCare Manitoba, 675 McDermot Ave, Winnipeg, MB, Canada R3E 0V9; Section of Oncology, Department of Internal Medicine, University of Manitoba, 820 Sherbrook Street, Winnipeg, MB, Canada R3A 1R9; Section of Oncology, Department of Internal Medicine, University of Manitoba, 820 Sherbrook Street, Winnipeg, MB, Canada R3A 1R9; Department of Internal Medicine, Rady Faculty of Health Sciences, University of Manitoba, 409 Taché Avenue, Winnipeg, MB, Canada R2H 2A6; Section of Cardiology, Department of Internal Medicine, University of Manitoba, 409 Taché Avenue, Winnipeg, MB, Canada R2H 2A6; Department of Cardiovascular Medicine, Mayo Clinic, 200 First Street SW, Rochester, MN 55905, USA

**Keywords:** glp1 receptor agonist, Anthracycline, Cardiotoxicity, Cardio-oncology, Diabetes

## Abstract

**Aims:**

Glucagon-like peptide-1 receptor agonists (GLP-1 RAs) reduce major adverse cardiovascular events in diabetics. This study aimed to investigate the cardiac, cancer, and overall mortality effects of GLP-1 RAs in diabetics treated with anthracyclines.

**Methods and results:**

We retrospectively identified 1384 diabetics treated with anthracyclines from 2013 to 2023; 56 received GLP-1 RAs between their first and last dose of anthracycline. These patients were propensity score matched 4:1 to 224 controls without concurrent GLP-1 RA use. Because stage was not available for 10 of the 56 patients, a repeat analysis was conducted with additional matching for cancer stage in 46 cases and 92 controls. Major endpoints were rate of all-cause and cancer-related and cardiovascular disease (CVD)–related mortality, no evidence of disease (NED), and hospitalization for CVD including heart failure (HF), over 5 years. Survival analyses were conducted using cause-specific hazards Cox regression. Patients on GLP-1 RAs had a significantly lower 5 year overall mortality (HR 0.46, CI 0.24–0.87, *P* = 0.007) and cancer-related mortality (HR 0.42, CI 0.19–0.91, *P* = 0.03). This mortality risk reduction remained significant even when accounting for covariates (HR 0.39, CI 0.20–0.75, *P* = 0.002). The percentage of patients with NED was higher in the GLP-1 RA than control group (60.9% vs. 38.0%, *P* = 0.007). A decline in left ventricular ejection fraction was seen in controls but not in patients on GLP-1 RAs. There was no significant difference in CVD hospitalization; the rate of HF-related hospitalization was very low overall.

**Conclusion:**

GLP-1 RAs may offer survival benefits and better cancer- and cardiac function-related outcomes in diabetics undergoing anthracycline chemotherapy.

## Graphical Abstract

**Graphical Abstract:**
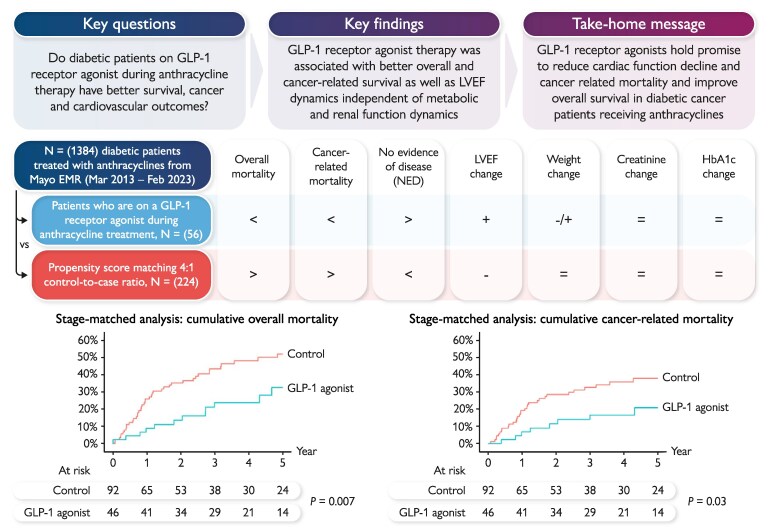

## Introduction

Diabetes has been shown to independently influence the risks and outcomes of cancer.^1^ In type 2 diabetes mellitus (T2DM), insulin resistance leads to hyperglycaemia and hyperinsulinaemia, which are recognized risk factors for cancer development.^2–4^ Furthermore, hyperglycaemia and hyperinsulinaemia have been shown to impact cancer progression and aggressiveness. Yet, diabetic patients are sometimes offered less aggressive cancer treatments due to concerns for increased cardiovascular, renal, and neuropathic complications.^5^ They also have more frequent infections, increased surgical complications, and increased chemotherapy toxicity.^6–8^

Glucagon-like peptide-1 receptor agonists (GLP-1 RAs) mimic GLP-1, a protein expressed in various tissues, including cardiomyocytes. They exert antidiabetic effects via post-prandial stimulation of insulin secretion and promotion of satiety, gluconeogenesis, and glycogenolysis.^9^ GLP-1 RAs exert cardio- and vaso-protective as well as anti-inflammatory effects through multiple mechanisms.^10,11^ The multifaceted benefits of GLP-1 RAs could be of high significance in the cancer population.^11^ Indeed, evidence for the oncological benefit of GLP-1 RAs has been emerging as well as the cardiovascular outcomes of cancer patients.^12-15^

Anthracyclines are used in the treatment of various cancers but are known for their association with cardiovascular toxicity and major adverse cardiovascular events (MACE).^16^ There are no standardized therapies for the prevention and treatment of anthracycline-induced cardiotoxicity (AIC). Beta blockers, angiotensin-converting enzyme (ACE) inhibitors, angiotensin receptor blockers (ARBs), sodium glucose co-transporter-2 (SGLT-2) inhibitors, and dexrazoxane have been advocated as preventive strategies, but not without debate.^17–19^ GLP-1 RAs have not been systematically studied as a potential cardioprotective agent for AIC. This study aimed to investigate the cardiac, cancer, and overall mortality outcomes of GLP-1 RAs in T2DM patients undergoing treatment with anthracyclines.

## Methods

**Figure 1:**
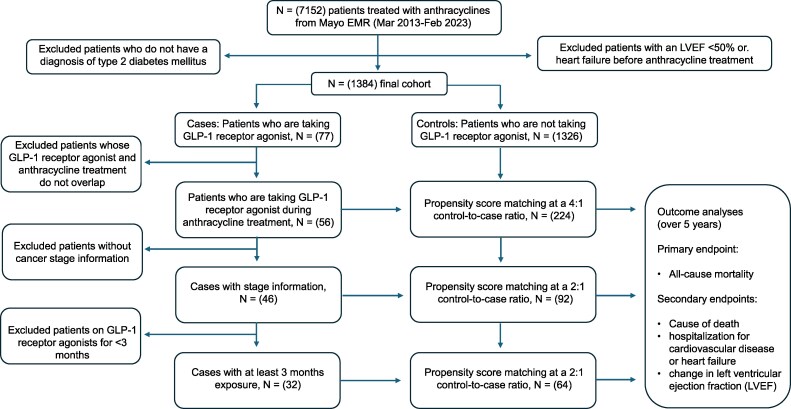
Study design flowchart. We initially identified 7152 patients treated with anthracyclines from 2013 to 2023. After the exclusion criteria, there were 1384 patients remaining. Of these, 56 patients were concurrently taking glucagon-like peptide-1 receptor agonists (GLP-1 RAs) with anthracyclines. Controls were matched to a 4:1 ratio by age, sex, obesity, Charlson score, and concurrent use of SGLT2 inhibitors, angiotensin-converting enzyme (ACE) inhibitors or angiotensin receptor blockers (ARB), metformin, and statins. The matching procedure was repeated for patients on GLP-1 RAs with defined stage or on therapy a minimum of 3 months.

The study was approved by the Institutional Review Board as a minimal risk study, meeting exempt criteria as defined by federal regulation 45 CFR 46. A cohort of patients treated with anthracyclines from 2013 to 2023 were extracted from the Mayo Electronic Medical Record (EMR). Patients included in the study were age ≥18, had T2DM, and had been diagnosed with cancer. The cohort was then stratified into cases and controls based on concurrent use and non-use, respectively, of a GLP-1 RA with anthracycline treatment (*Figure 1*). Patients on GLP-1 RA (exenatide, lixisenatide, oral semaglutide, dulaglutide, exenatide ER, liraglutide, and semaglutide) were counted only if they started the medication before or simultaneously with anthracycline initiation. Descriptive statistics were compiled for patient characteristics in each group. The primary outcome of interest was all-cause mortality over 5 years. Secondary outcomes were cause of death, hospitalization for cardiovascular disease (CVD) or heart failure (HF) over 5 years and change in left ventricular ejection fraction (LVEF) per year and over 5 years. CVD hospitalization included hospital diagnoses of atrial fibrillation, acute HF, coronary artery disease, and valvular disease. Cause of death was adjudicated via chart review and defined as cardiovascular, infectious, malignant, or other. The baseline date for patient follow-up was defined as the initial date of anthracycline treatment. Baseline comorbidities needed to be established before the baseline date, and baseline medications were defined as medications the patient started before the baseline date and continued at least 7 days after the baseline date.

Propensity scores were used to match patients in case and control groups. The probability of being in the treatment group was calculated using logistic regression, and the propensity score was estimated for the average treatment effect on the treated (ATT). Covariates included in the propensity score model were age, sex, obesity, Charlson Comorbidity Index, and concurrent use of SGLT-2 inhibitors, ACE inhibitors, or ARBs, metformin, and statins. Patients were matched using a 4:1 control/treated ratio by minimizing the sum of absolute pairwise distances.^20^ Balance between groups was assessed using standardized mean differences (SMD), with all covariates achieving an SMD <0.10, indicating adequate balance.

Kaplan–Meier methods were used to generate a cumulative incidence curve of 5 year all-cause mortality, and differences between groups were tested using a log-rank test. For secondary outcomes, cumulative incidence curves were generated and compared using Gray’s test to account for the competing risk of death. In the case of multiple outcomes, the curves represent the time to first event. The association between GLP-1 RA use and each outcome was evaluated using cause-specific Cox proportional hazards models. Univariate Cox models were first fit for mortality using baseline comorbidities and medication use. A multivariable Cox model was then constructed including all covariates with *P* < 0.10 in univariate analyses. Robust variance estimators were used in all Cox models to account for clustering within matched sets.

These analyses were repeated in the subset of patients with known cancer stage, using a 2:1 control/treated matching ratio due to the smaller number of available controls. The analyses were further repeated after subsetting to patients with a minimum of 3 months of GLP-1 RA treatment. In the final subset, 2.5 year mortality was also assessed.

Linear mixed-effects models were used to analyse longitudinal changes in body weight, creatinine, and HbA1c accounting for repeated measurements and the clustering of matched patients. Weight was modelled as a function of time, GLP-1 RA use, and their interaction. Random intercepts were included for the grouping variable identifying matched patients as well as the individual patients nested within the matching group variable to account for between-group variability. Marginal predictions were then plotted against time to visualize the cohort specific trajectories over time. These models were fit using both a linear variable for time as well as a non-linear estimated using a penalized spline. An interrupted time series was used to model weight change while on GLP-1 followed by the weight change after GLP-1 was stopped.

LVEF data was filtered to only include patients with a baseline transthoracic echocardiogram (TTE) within 1 year of their baseline date. Change in LVEF per year was calculated from the baseline TTE to the most recent follow-up TTE; change in LVEF over a year was calculated for patients who had a follow-up TTE within 1 year of baseline date. Comparisons were conducted via the Student’s *t*-test, and patients were not matched for this analysis. An adjusted comparison of change in LVEF over time was performed using the linear mixed-effects model; patients were matched for this analysis.

Cancer-related outcomes were abstracted by chart review for all patients. The main outcome parameter was the percentage of patients with now evidence of disease (NED) over the follow-up period from completion of cancer therapy to last oncological visit on record. Group comparisons were made by Gray’s test.

Significance for all tests was defined as *P* < 0.05. Data manipulation and statistical testing were done in R Statistical Software, version 4.4.1. Differences in baseline characteristics were assessed on the matched population using conditional logistic regression.

## Results

### Overall cohort

A total of 1384 patients with diabetes were identified who were treated with anthracyclines between 2013 and 2023. Of these, 56 patients (4.0%) were taking a GLP-1 RA concurrently with anthracycline treatment. These patients were propensity score-matched 1:4 to 224 control patients (*Figure 1*). Overall characteristics of the GLP-1 RA and matched control group were largely similar, only difference being that patients on GLP-1 RA were more likely to be simultaneously taking sulfonylureas (see Supplementary material online, *Table S1*). The GLP-1 RA group had a significantly lower risk of 5 year overall mortality (18/56 [32.1%] vs. 113/224 [50.4%]; HR 0.54, CI 0.31–0.92, *P* = 0.03) and cancer-related mortality (10/56 [17.9%] vs. 72/224 [32.1%]; HR 0.47, CI 0.24–0.92, *P* = 0.03) (see Supplementary material online, *Figure S1*). This mortality risk reduction remained significant even when accounting for CVD, other comorbidities, concurrent use of other antiglycaemics, or cardiovascular medications (HR 0.48, 95% CI 0.28–0.82, *P* = 0.007; Supplementary material online, *Table S2*). Malignancy was the leading cause of death in both groups (17.9% vs. 32.1%, *P* = 0.04, Supplementary material online, *Table S3*), and the median time to death was 2.0 years in the GLP-1 RA group and 1.1 years in the control group (Wilcox test *P* = 0.08). The percentage of patients with NED over the follow-up period was significantly higher in the GLP-1 RA than control group (58.9% vs. 39.7%, *P* = 0.01).

There was no significant difference in overall risk of CVD hospitalization comparing the two groups (27.9% vs. 26.8%; HR 0.86, CI 0.47–1.58, *P* = 0.62, Supplementary material online, *Table S4*). In linear mixed-effects models, the main effect of GLP-1 RAs was not significant for body weight. The cohort-by-time interaction paradoxically indicated a small increase in body weight of 0.91 (CI 0.73–1.10) kg per year in the GLP RA group. This phenomenon emerged after the end date of GLP-1 RA therapy. Using an interrupted time series model, it was estimated that patients lost on average 1.05 (CI 0.72–1.38) kg/year while on GLP-1 RA and gained an average of 2.20 (CI 1.86–2.54) kg/year after cessation in the case group. Creatinine showed a significant effect (0.36, CI 0.07–0.66) of GLP-1 RA use with no significant interaction, consistent with a stable between-group difference over time. For HbA1c, neither the GLP-1 RA effect nor the interaction was statistically significant (see Supplementary material online, *Figures S2–S4*).

### Stage-matched cohort

**Figure 2:**
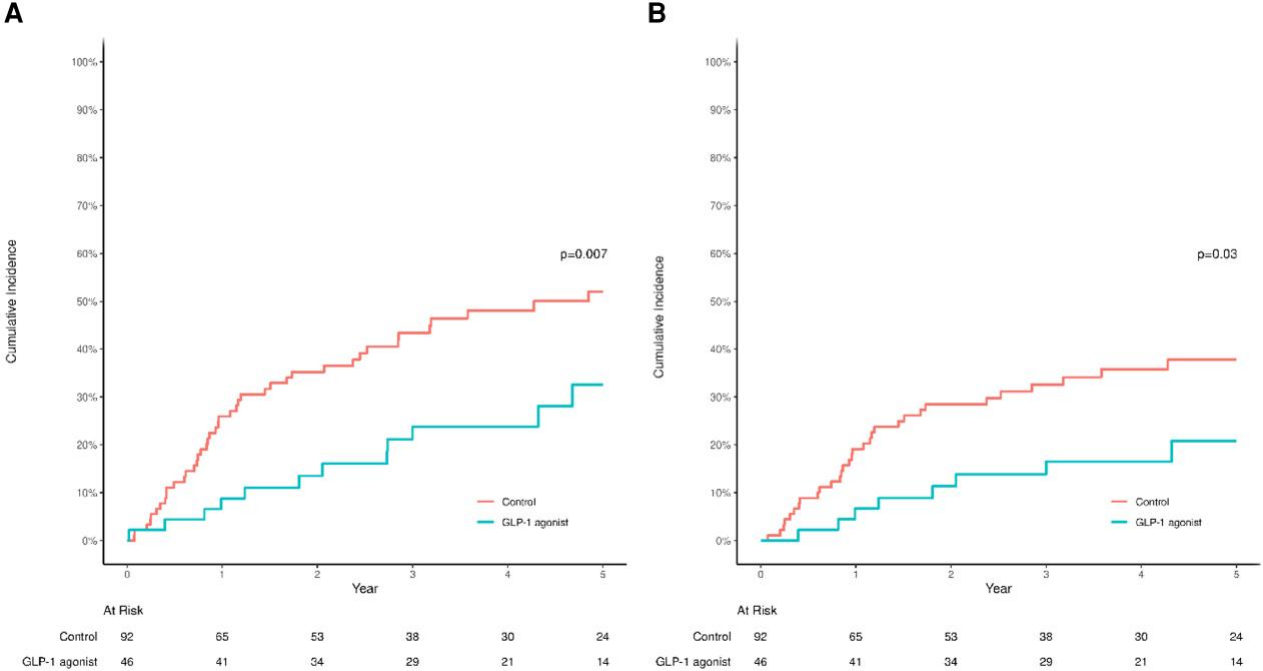
Cumulative 5-year mortality. Cumulative incidence curves of 5 year all-cause mortality (*A*) and death due to malignancy (*B*) in diabetic patients treated with anthracyclines was compared for those concurrently taking (teal) or not taking (red) a GLP-1 RAs.

Of the 56 patients of the overall cohort, stage information was available on 46, and these patients were matched 1:2 to 92 control patients. As outlined in *Table 1*, the two groups were well matched, only difference being that patients on GLP-1 agonists were more likely to be simultaneously taking sulfonylureas (*Table 1*). In the cumulative event analyses, patients in GLP-1 RA group again had significantly lower risk of 5 year overall mortality (14/46 [30.4%] vs. 46/92 [50.0%]; HR 0.46, CI 0.24–0.87, *P* = 0.007) and cancer-related mortality (8/46 [17.4%] vs. 31/92 [33.7%]; HR 0.42, CI 0.19–0.91, *P* = 0.02) (*Figure 2*). This mortality risk reduction remained significant even when accounting for covariates (HR 0.39, CI 0.20–0.75, *P* = 0.002; *Table 2*). Malignancy was the leading cause of death in both groups (17.4% vs. 33.7%; *P* = 0.04, *Table 3*), and the median time to death from cancer treatment was 2.4 years in the GLP-1 RA group and 1.0 years in the control group (Wilcox test *P* = 0.11). The percentage of patients without NED over the follow-up period was significantly higher in the GLP-1 RA than control group (60.9% vs. 38.0%, *P* = 0.007).

**Table 1:** Matched patient baseline characteristics

	GLP-1 RAs (*n* = 46)	Controls (*n* = 92)	*P* ^a^
**Age (mean, SD)**	64.3 (10.0)	64.8 (10.8)	0.77
**Sex,** f**emale (*n*, %)**	30 (65.2)	61 (66.3)	0.89
**Malignancy (*n*, %)**			
** Lymphoma**	15 (32.6)	32 (34.8)	0.07
** Leukaemia**	1 (2.2)	4 (4.3)	
** Breast**	16 (34.8)	28 (30.4)	
** Sarcoma**	8 (17.4)	3 (3.3)	
** Other/unspecified**	6 (13.0)	25 (27.2)	
**Baseline CVD (*n*, %)**	24 (52.2)	38 (41.3)	0.21
** CAD**	11 (23.9)	21 (22.8)	0.89
** CHF**	0 (0.0)	0 (0.0)	—
** AF**	3 (6.5)	9 (9.8)	0.51
** Valvular disease**	16 (34.8)	25 (27.2)	0.35
**Other comorbidities (*n*, %)**			
** HTN**	34 (73.9)	73 (79.3)	0.47
** HLD**	33 (71.7)	69 (75.0)	0.67
** Tobacco** u**se**	20 (43.5)	46 (50.0)	0.48
** Obesity**	39 (84.8)	77 (83.7)	0.87
** CKD**	12 (26.1)	19 (20.7)	0.46
**Concurrent antiglycaemics (*n*, %)**			
** Metformin**	28 (60.9)	53 (57.6)	0.71
** Sulfonylureas**	15 (32.6)	15 (16.3)	**0**.**04**
** Insulin**	26 (56.5)	39 (42.4)	0.13
** SGLT2i**	5 (10.9)	9 (9.8)	0.84
**Concurrent CVD** m**edications (*n*, %)**			
** BB**	17 (37.0)	35 (38.0)	0.90
** ACEi/ARB**	28 (60.9)	51 (55.4)	0.51
** ARNi**	0 (0)	0 (0)	—
MRA	0 (0)	6 (6.5)	0.99
** Diuretics**	9 (19.6)	19 (20.7)	0.88
** CC blocker**	12 (26.1)	24 (26.1)	1.00
** Statin**	32 (69.6)	64 (69.6)	1.00
**GLP-1 RA**			
** Exenatide**	5 (10.9)	—	—
** Lixisenatide**	1 (2.2)	—	—
** Oral** s**emaglutide**	1 (2.2)	—	—
** Dulaglutide**	12 (26.1)	—	—
** Exenatide ER**	4 (8.7)	—	—
** Liraglutide**	16 (34.8)	—	—
** Semaglutide**	7 (15.2)	—	—

ACEi, angiotensin-converting enzyme inhibitor; AF, atrial fibrillation; ARB, angiotensin II receptor blocker; ARNi, angiotensin receptor-neprilysin inhibitor; BB, beta blocker; CAD, coronary artery disease; CC, calcium channel; CHF, congestive heart failure; CKD, chronic kidney disease; CVD, cardiovascular disease; HLD, hyperlipidaemia; HTN, hypertension; MRA, mineralocorticoid receptor antagonist; SD, standard deviation; SGLT2i, sodium glucose cotransporter 2 inhibitor.

^a^Conditional logistic regression was used to estimate the *P*-values while accounting for the matching.

**Table 2:** Risk factor analysis of all-cause mortality

	Univariate			Multivariable^a^		
	HR	CI	*P*	HR	CI	*P*
**Baseline CVD^b^**	1.74	1.12–2.71	**0**.**01**			
** CAD**	1.81	1.13–2.89	**0**.**01**	1.46	0.77–2.76	0.17
** AF**	2.17	1.04–4.56	**0**.**04**	1.12	0.46–2.73	0.78
** Valvular** d**isease**	1.80	1.04–3.12	**0**.**03**	1.54	0.83–2.84	0.17
**Other comorbidities**						
** HTN**	1.67	0.89–3.16	0.11	—	—	—
** HLD**	1.52	0.81–2.84	0.19	—	—	—
** Tobacco use**	0.95	0.60–1.51	0.84	—	—	—
** CKD**	2.24	1.29–3.90	**0**.**004**	2.00	1.05–3.79	**0.02**
**Antiglycaemics**						
** Sulfonylureas**	0.82	0.41–1.65	0.75	—	—	—
** Insulin**	1.46	0.85–2.53	0.17	—	—	—
** GLP-1 RAs**	0.46	0.26–0.81	**0**.**007**	0.39	0.20–0.75	**0.002**
**Concurrent CVD medications^c^**						
** BB**	1.26	0.68–2.33	0.47	—	—	—
**Mineralocorticoid**	1.48	0.48–4.61	0.50	—	—	—
** Diuretics**	1.96	1.04–3.71	**0**.**04**	1.28	0.66–2.50	0.49
** CC** b**locker**	1.47	0.77–2.79	0.24	—	—	—

^a^Variables with *P* < 0.10 in univariate regression were included in the multivariable model.

^b^CHF was not run due to having no patients in the sample. ARNi was not run due to having only 1 patient in the sample.

^c^ARNi was not run due to having only 1 patient in the sample.

**Table 3:** Causes of death

	GLP-1 RAs (*n* = 46)	Control (*n* = 92)
**Cardiovascular**	0 (0)	2 (2.2)
**Infectious/septic**	0 (0)	3 (3.3)
**Malignancy^a^**	8 (17.4)	31 (33.7)
**Other^b^**	1 (2.2)	3 (3.3)
**Unknown**	5 (10.9)	7 (7.6)

^a^Includes hospice-qualifying diagnoses and complications related to malignancy.

^b^Includes other organ failure such as acute kidney injury or respiratory failure.

There was no significant difference in overall risk of CVD hospitalization comparing the two groups (27.6% vs. 20.6%; HR 1.16, CI 0.54–2.48, *P* = 0.72, *Table 4*). When subtracting baseline LVEF from the LVEF on follow-up and dividing that difference by the total time between measurements, a decline in LVEF was seen in controls but not in patients on GLP-1 RA (−17.6 +/− 66.1 vs./21.7 +/− 111.3, *P* = 0.04). Additional echocardiographic parameters, including global longitudinal strain, stroke volume index, and cardiac index, were available for a limited subset of patients, with no statistically or clinically significant differences observed between groups (*Table 5*). Diastolic function indices such as E/e′, e′ velocities, and left atrial volume index were also similar and fell within the normal or indeterminate ranges for most patients. RV function and chamber size measurements showed no consistent between-group differences.

**Table 4:** Cardiovascular disease hospitalizations

	GLP-1 RAs (*n* = 46)	Control (*n* = 92)
**CVD hospitalizations (*n*, %)^a^**	11 (23.9)	17 (18.5)
** Atrial fibrillation**	1 (2.2)	4 (4.3)
** Acute heart failure**	4 (8.7)	6 (6.5)
** Coronary artery disease**	2 (4.3)	7 (7.6)
** Valvular disease**	4 (8.7)	0 (0)
**5 year composite cumulative risk (%)^b^**	27.6	20.6

^a^Atrial fibrillation included both paroxysmal and permanent atrial fibrillation. Acute heart failure included both systolic and diastolic heart failure. Valvular disease included rheumatic, non-rheumatic, native valve, and prosthetic valve disease.

^b^Composite cumulative risk Gray’s test *P*-value = 0.57.

**Table 5:** Echocardiographic parameters by GLP-1 agonist use for the unmatched population (***n* = number of patients with complete data included in the analyses)**

	GLP-1 RAs		Control		*P* value
	*n*	Mean (SD)	N	Mean (SD)	
LV systolic function					
Global longitudinal strain	8	−18.4 (3.5)	71	−18.6 (6.9)	0.91
Stroke volume index	36	40.0 (8.9)	1136	43.3 (9.3)	0.04
Cardiac output	35	7.0 (1.6)	1114	6.5 (1.6)	0.11
Cardiac index	35	3.3 (0.7)	1102	3.3 (0.7)	0.72
LV diastolic function					
E/A ratio	34	0.9 (0.3)	1189	1.0 (0.4)	0.20
Septal e’	35	0.08 (0.02)	1199	0.09 (0.26)	0.82
Lateral e’	23	0.10 (0.02)	845	0.1 (0.03)	0.83
E/e’ ratio (average)	22	8.4 (2.5)	832	9.3 (3.0)	0.19
Mitral inflow deceleration time	31	223 (70)	1110	215 (52)	0.35
LAVI	33	27.5 (7.6)	1157	29.4 (8.5)	0.22
LV size and structure					
LV mass index	34	88.6 (22.5)	1219	86.8 (20.1)	0.61
RWT	34	0.46 (0.09)	1221	0.43 (0.08)	0.08
RV systolic and diastolic function					
TAPSE	19	22.1 (4.0)	475	23.2 (4.5)	0.27
RV S’	25	0.15 (0.03)	771	0.19 (1.11)	0.89
RV FAC	4	41.0 (11.6)	41	42.0 (8.5)	0.84
RV strain	1	−37.0	73	−26.4 (4.7)	—

E/A, early-to-late mitral inflow velocity ratio; E/e′, mitral inflow to annular velocity ratio; e′, early diastolic mitral annular velocity; FAC, fractional area change; GLP-1, glucagon-like peptide-1; LAVI, left atrial volume index; LV, left ventricle; LVEF, left ventricular ejection fraction; LVMI, left ventricular mass index; RWT, relative wall thickness; RV, right ventricle; RV S′, right ventricular systolic annular velocity; SD, standard deviation; SVI, stroke volume index; TAPSE, tricuspid annular plane systolic excursion.

### Additional subgroup analyses

When the inclusion criteria for analysis were modified to include only patients on GLP-1 RAs for at least 3 months prior to the start of anthracycline therapy, 31 patients were in the intervention group. The median time from first GLP-1 RA prescription to anthracycline initiation in this subgroup was 2.4 years (IQR 1.3–3.8 years). These were propensity score-matched 1:2 to 62 control patients. The overall characteristics of this GLP RA and matched control group are outlined in Supplementary material online, *Table S5*. Both cohorts were again well matched without significant differences. The GLP-1 RA group had significantly lower risk of 2.5 year overall mortality (12/31 [38.7%] vs. 28/62 [45.2%]; HR 0.46, CI 0.21–0.99, *P* = 0.049) and cancer-related mortality (7/31 [22.6%] vs. 20/62 [32.3%]; HR 0.56, CI 0.22–1.41, *P* = 0.23) (see Supplementary material online, *Figure S5*). This risk was no longer significant after accounting for CVD, other comorbidities, concurrent use of other antiglycaemics or cardiovascular medications (HR 0.47, 95% CI 0.18–1.20, *P* = 0.08; Supplementary material online, *Table S6*). Malignancy was the leading cause of death in both groups (22.6% vs. 32.3%; *P* = 0.33, Supplementary material online, *Table S7*), and the median time to death from cancer treatment was 2.1 years in the GLP-1 RA group and 1.2 years in the control group (Wilcox test *P* = 0.09). There was no significant difference in overall risk of CVD hospitalization comparing the two groups (31.8% vs. 28.0%; HR 0.91, CI 0.39–2.14, *P* = 0.82, Supplementary material online, *Table S8*).

## Discussion

This is one of the first studies to demonstrate that GLP-1 RA use in patients with T2DM undergoing anthracycline treatment is associated with a significant reduction in 5 year all-cause and cancer-related mortality as well as a significant increase in complete remission rates over the follow-up period, compared to those not using these medications. LVEF dynamics over time were also more favourable in the GLP-1 RA group. These benefits were independent of a significant reduction in weight, HbA1c, or creatinine over time. Overall these findings suggest that GLP-1 RA may offer oncological and cardiovascular benefits that warrant further exploration in prospective trials.

One of the prime objectives of this study was to assess if diabetic patients with malignancy undergoing anthracycline-based chemotherapy on GLP-1 RA have a lower risk of AIC. Anthracyclines lead to cardiac dysfunction and remodelling through several mechanisms, including disruption of mitochondrial function with impairment of ATP production, increase in oxidative stress, alteration in calcium homeostasis, interference with DNA repair, and activation of apoptotic pathways, inflammatory responses and eventually fibrosis.^21–24^ GLP-1 RAs antagonize these mechanistic pathways and may henceforth mitigate anthracycline cardiotoxicity.^25–30^ In keeping with this theory, in this study a decline in LVEF was not seen in patients on GLP-1 RAs while it was present in those not on these medications during anthracycline therapy in support of cardioprotective effects of GLP-1 RAs. Clinical events such as HF hospitalization were too low to allow for the demonstration of any notable benefit. Preliminary data by Chiang *et al.* do indicate though that GLP-1 RAs are also able to reduce major adverse cardiovascular events (MACE) in diabetic patients with breast cancer and T2DM treated with anthracyclines if on GLP-1 RA.^15^ Thus, a beneficial cardiovascular role for GLP-1 RA in cancer patients is emerging including protection against cancer therapy-related toxicities.

The data by Chiang *et al.* also show a reduction in all-cause mortality among diabetic breast cancer patients on GLP-1 RA therapy.^15^ This was also observed in our study; in fact, the most striking observation was the reduction in mortality risk with GLP-1 RAs, overall and cancer-related. This constellation and the finding that the percentage of patients with NED over the follow-up period was significantly higher in patients receiving GLP-1 RAs points to a predominant oncological benefit. This is contrast to the concerns of a pro-oncogenic effect of GLP-1 RA leading to black box warning, which were raised by preclinical rodent data and meta-analyses of randomized clinical trials indicating a possible increased risk of thyroid cancer (OR 1.55; 95% CI, 1.05–2.27).^31^ However, not all meta-analyses reached this conclusion. In fact, a recent systemic review and meta-analysis found that GLP-1 RAs have little to no effect on thyroid, pancreatic, breast, or kidney cancer risk (moderate certainty, low certainty for other cancer types).^32^ A large target trial emulation study of 86 632 matched adults with obesity/overweight found a 17% lower overall cancer risk associated with GLP-1 RA use (HR 0.83; 95% CI, 0.76–0.91).^33^ A separate EMR-based study of >1.6 million patients with T2DM found that GLP-1 RA use compared with insulin was associated with significant risk reductions in 10 of 13 obesity-associated cancers.^13^ Incorporating both randomized controlled trials (RCTs) and observational studies (*n* = 3 960 974), a very recent meta-analysis concluded on a 30% lower overall risk of obesity-related cancers (RR 0.70; 95% CI, 0.54–0.89).^34^ What remains as a more consistent signal is a potentially increased kidney cancer risk that has been noted across multiple studies (HR ∼1.38–1.54), particularly in younger patients and those with overweight (BMI 27–29.9).^35^ It is important to point out that all current evidence is limited by short follow-up in RCTs, detection and prescription biases in observational studies, and the absence of prospective trials, specifically designed and powered for cancer outcomes.

In terms of mortality benefits, a TriNetX database study of 55 808 patients with active cancer and T2DM found that GLP-1 RA use was associated with reduced all-cause mortality compared to metformin (HR 0.875; 95% CI, 0.778–0.985).^36^ An even greater mortality benefit was seen in patients who were newly started on GLP-1 RAs near cancer treatment initiation (HR 0.786; 95% CI, 0.662–0.934); the risk of hospitalization, sepsis, and MACE was also lower.^36^ A Medicare-based study of older adults with cancer and type 2 diabetes showed that GLP-1 RA users had a significantly lower mortality than DPP4i users; the benefit was particularly evident for colorectal, lung, and breast cancers, while mortality was comparable to SGLT2i users.^37^ Last but not least, a very recent retrospective analysis using data from the University of California Health Data Warehouse on 6871 patients with colorectal cancer found a more than 50% lower 5 year mortality in GLP-1 RA users vs. non-users (15.5% vs. 37.1%, OR = 0.38, 95% CI: 0.21–0.64, *P* < 0.05).^38^ Thus, our study is very much in agreement with the results of these studies and adds to the emerging oncological benefit of GLP-1 RAs on the background of the reviewed oncological concerns.

Mechanistically, preclinical studies using *in vitro* cancer cell models and *in vivo* mouse models of liver, colorectal, pancreatic, and lung cancer have demonstrated that GLP-1 RAs inhibit tumour progression via several potential mechanisms.^39–43^ For instance, GLP-1 RAs have been shown to inhibit the growth and induce apoptosis of breast cancer cells via increase in cAMP levels and activity, even though these cancer cells lack classic GLP-1 receptors.^44^ Other studies found that GLP-1 RAs restore natural killer (NK) cell function, a key defence against development of malignancies, which is exhausted in patients with obesity.^45^ Other studies did not find a beneficial effect of GLP-1 RAs on NK cell function but rather CD11c+ dendritic cells and CD8+ T cells, suggesting they may enhance the acquired antitumour immune response.^46^ GLP-1 RAs also modulate macrophage behaviour, specifically inhibiting pro-inflammatory M1 polarization and promoting anti-inflammatory M2 phenotypes.^47,48^ They inhibit the IL-6/STAT3 pathway and NF-κB signalling, reducing pro-inflammatory cytokine production (TNF-α, IL-6) and mitigating oxidative stress.^49^ Last but not least, the oncological benefit of GLP-1 RAs might be mediated by the reduction of chronic hyperinsulinaemia. Multiple insulin-regulated pathways have been implicated in tumour resistance and metastasis,^50–52^ and insulin and its analogs have also been shown to significantly increase chemoresistance to anthracyclines by antagonizing anthracycline-induced apoptosis.^53,54^ Concordantly, insulin therapy is associated with worse outcomes in diabetic patients with breast cancer, including increased overall mortality, increased breast cancer-specific mortality, and increased recurrence rates.^55^

The discourse on GLP-1 RA is reminiscent of the discussion of oncological and survival benefits of SGLT-2 inhibitors in cancer patients. Gongora *et al.* showed that mortality over a median follow-up period of 1.5 years was 45% in patients not receiving vs. 9% in patients receiving SGLT-2 inhibitors (HR 4.7, 95% CI 1.5–15.1, *P* = 0005).^56^ Some of this benefit was attributed to improvement in CV outcomes; however, improved survival was seen also in studies with fewer CV events such as the one by Huang *et al.* who showed a significantly lower mortality compared to diabetics not on SGLT-2 inhibitors (adjusted HR = 0.35, 95% CI = 0.25–0.51) and non-diabetics (adjusted HR = 0.33, 95% CI = 0.23–0.49, *P* < 0.05 for both) over a median follow-up period of 3.4 years.^57^ The authors also discussed weight reduction and a related reduction in obesity-related malignancies. However, SGLT-2 inhibitors are not weight reduction drugs, and a more direct effect on cancer cells remained the most compelling hypothesis. Indeed, SGLT2 inhibitors have been shown to slow tumour growth in mouse models by promoting a fasting-like state and mitigating hyperinsulinaemia. In keeping, Bhatti *et al.* found that cancer patients undergoing anthracycline therapy on SGLT-2 inhibitors, compared to those who did not, not only had a lower all-cause mortality (7.5% vs. 11.9%, HR 0.67, 95% CI 0.61–0.74, *P* < 0.001) but also a lower rate of new metastatic cancer (4.5% vs. 7.6%, HR 0.66, 95% CI 0.58–0.75, *P* < 0.001) over 1 year of follow-up.^58^ Our study was not designed to directly evaluate the effects of GLP-1 RAs on cancer progression or aggressiveness or to compare different antidiabetic drugs. In agreement with the evolving landscape, the outlined studies, including ours, encourage further efforts to define how GLP-1 RAs influence tumour biology and assess treatment tolerability in cancer patients undergoing chemo- and other therapies. Comparative studies are warranted to evaluate the additive or synergistic effects of combining GLP-1 RAs with beta blockers, ACE inhibitors, or ARBs, statins, and SGLT-2 inhibitors.

### Strengths and limitations

The study’s strengths include its robust methodology with propensity score matching to minimize confounding and the comprehensive analysis of long-term outcomes, providing valuable insights into the potential benefits of GLP-1 RAs in T2DM patients undergoing anthracycline treatment. Several limitations of our study warrant consideration though. The small sample size of the GLP-1 RA group limits the generalizability of our findings and increases the potential for residual confounding. The retrospective design of the study precludes establishing causality between GLP-1 RA use and improved outcomes. The reliance on electronic medical records may have introduced selection bias, particularly in the identification of comorbidities and medication use. Additionally, medication adherence could not be directly confirmed, as our data captures prescribed medications rather than dispensed or administered doses. Future multicentre studies in larger cohorts are needed to confirm the impact of GLP-1 RA on survival outcome and recurrence of disease of cancer patients as well as cardiovascular outcomes. Ideally any future studies should include prospective data acquisition and most ideally take the form of a randomized controlled trial.

## Conclusions

Our findings suggest that GLP-1 RAs may confer better survival and cancer- and cardiac function-related outcomes in diabetic cancer patients undergoing anthracycline chemotherapy. Future studies are needed to define the mechanisms underlying this treatment benefit and prospective RCTs to confirm efficacy, safety, and tolerability of GLP-1 RAs as adjunctive therapies in the cancer population. Ultimately, a multidisciplinary approach remains essential for optimizing care and improving outcomes for patients with various types of malignancies, comorbidities, and cancer therapies.

## Supplementary Material

oeag109_Supplementary_Data

## Data Availability

The data generated and analysed in this study are not publicly available due to privacy or ethical restrictions but will be available from the corresponding author upon reasonable request and pending explicit approval of the study investigators and a signed data usage agreement between the participating institutions.
