# Supplementary material for: Glucagon-like peptide-1 receptor agonists are associated with cardiac, cancer- and mortality-related benefits in diabetic patients treated with anthracyclines
Source: Eur Heart J Open. 2026 Jun 25;6(4):oeag109. doi: 10.1093/ehjopen/oeag109 (PMC13384431; doi:10.1093/ehjopen/oeag109)
Supplement: oeag109_Supplementary_Data [file oeag109_supplementary_data.docx]

**SUPPLEMENTAL DATA**

Glucagon-like Peptide-1 Receptor Agonists Improve Survival in Diabetics Treated with Anthracyclines

Sunnia T. Chen et al.

**Figure S1: Cumulative Incidence of 5-Year Mortality Including Patients Where Cancer Stage is Unknown**


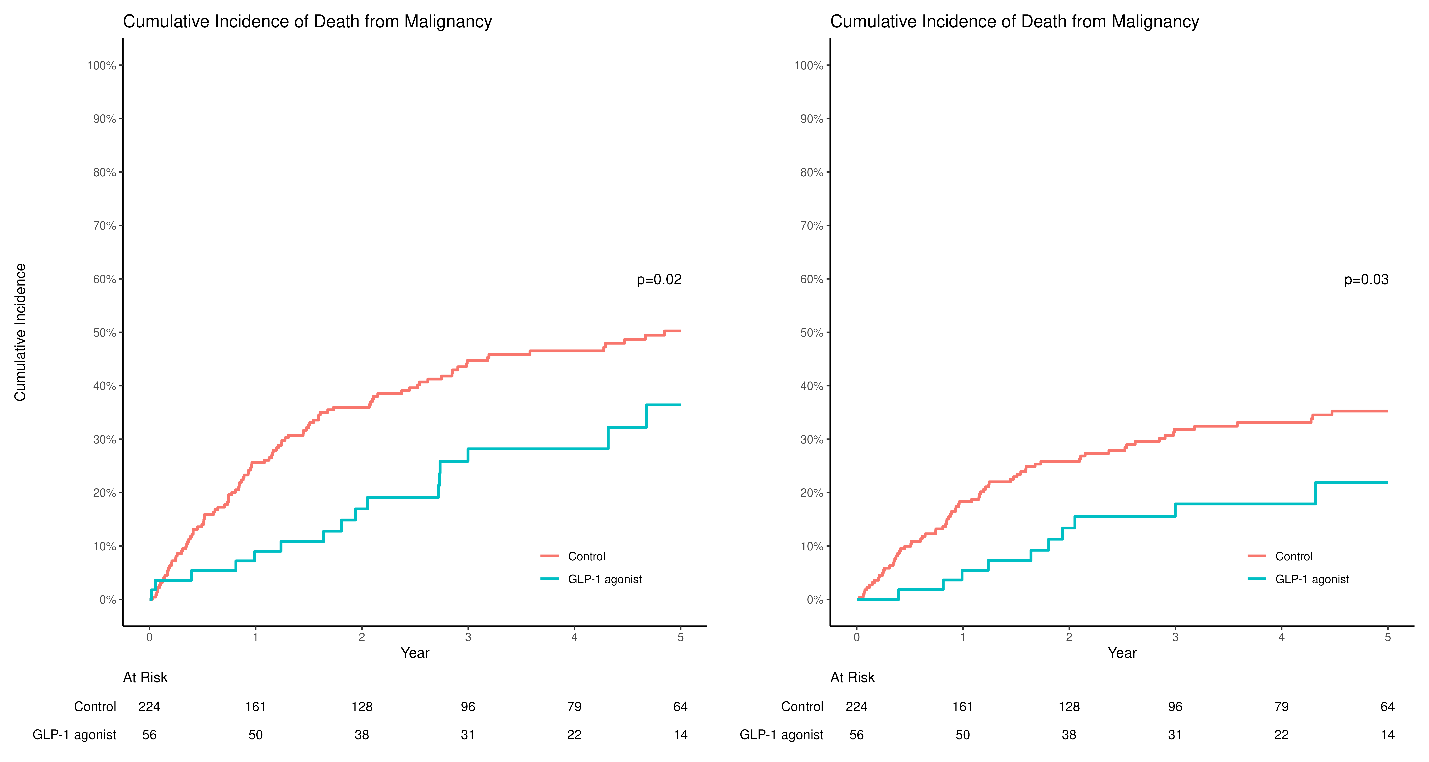


Cumulative incidence curves of 5-year all-cause mortality (left) and death due to malignancy (right) in diabetic patients treated with anthracyclines with (blue) or without (red) concomitant glucagon-like peptide-1 (GLP-1) receptor agonist therapy.

**Figure S2: Weight Changes Across Time Including Patients Where Cancer Stage is Unknown**

**
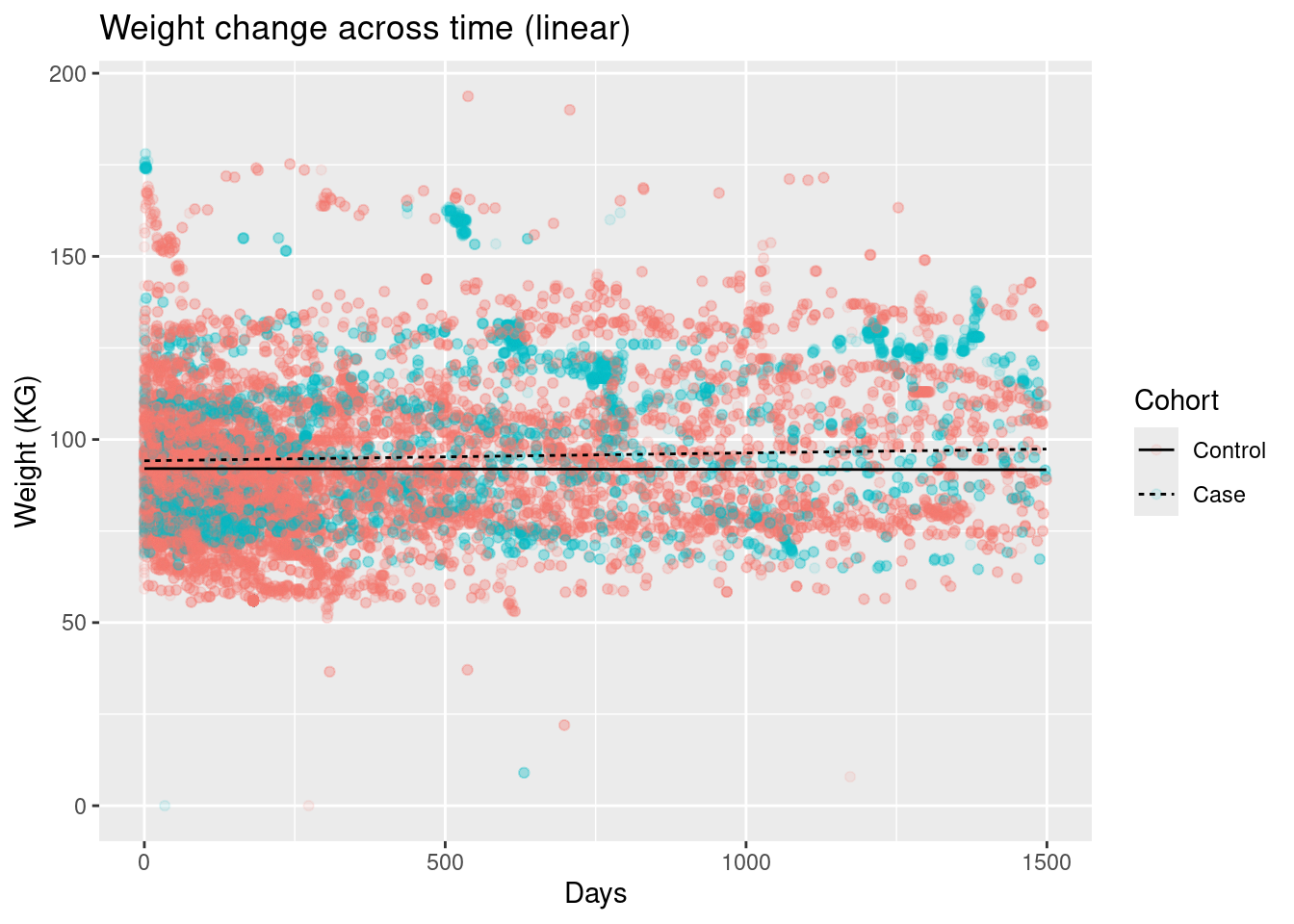

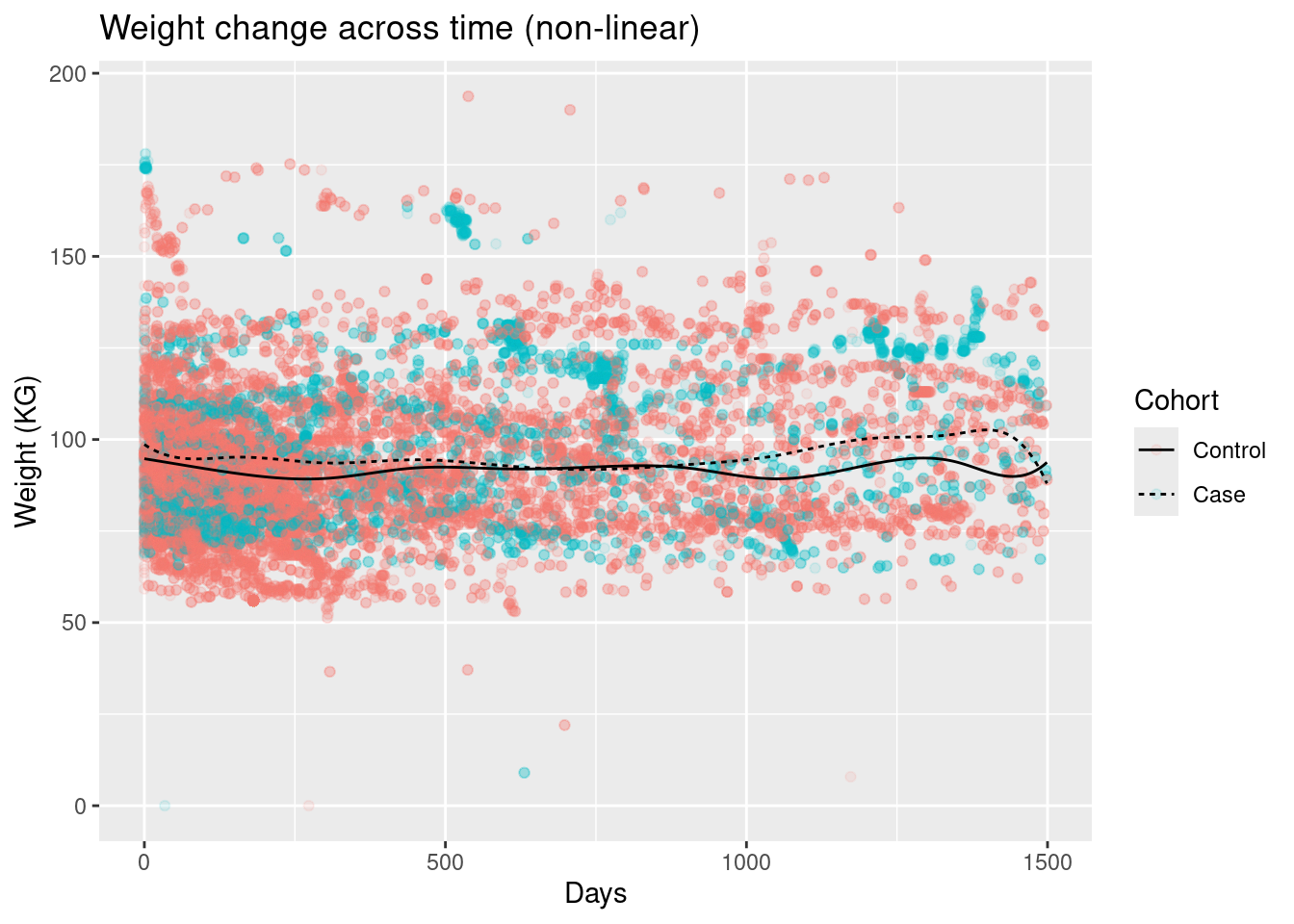
**

Changes in body weight (kg) over time in diabetic patients treated with anthracyclines with (dotted line) or without (solid line) concomitant glucagon-like peptide-1 (GLP-1) receptor agonist therapy.

**Figure S3: Creatinine Serum Concentration Changes Across Time Including Patients Where Cancer Stage is Unknown**

**
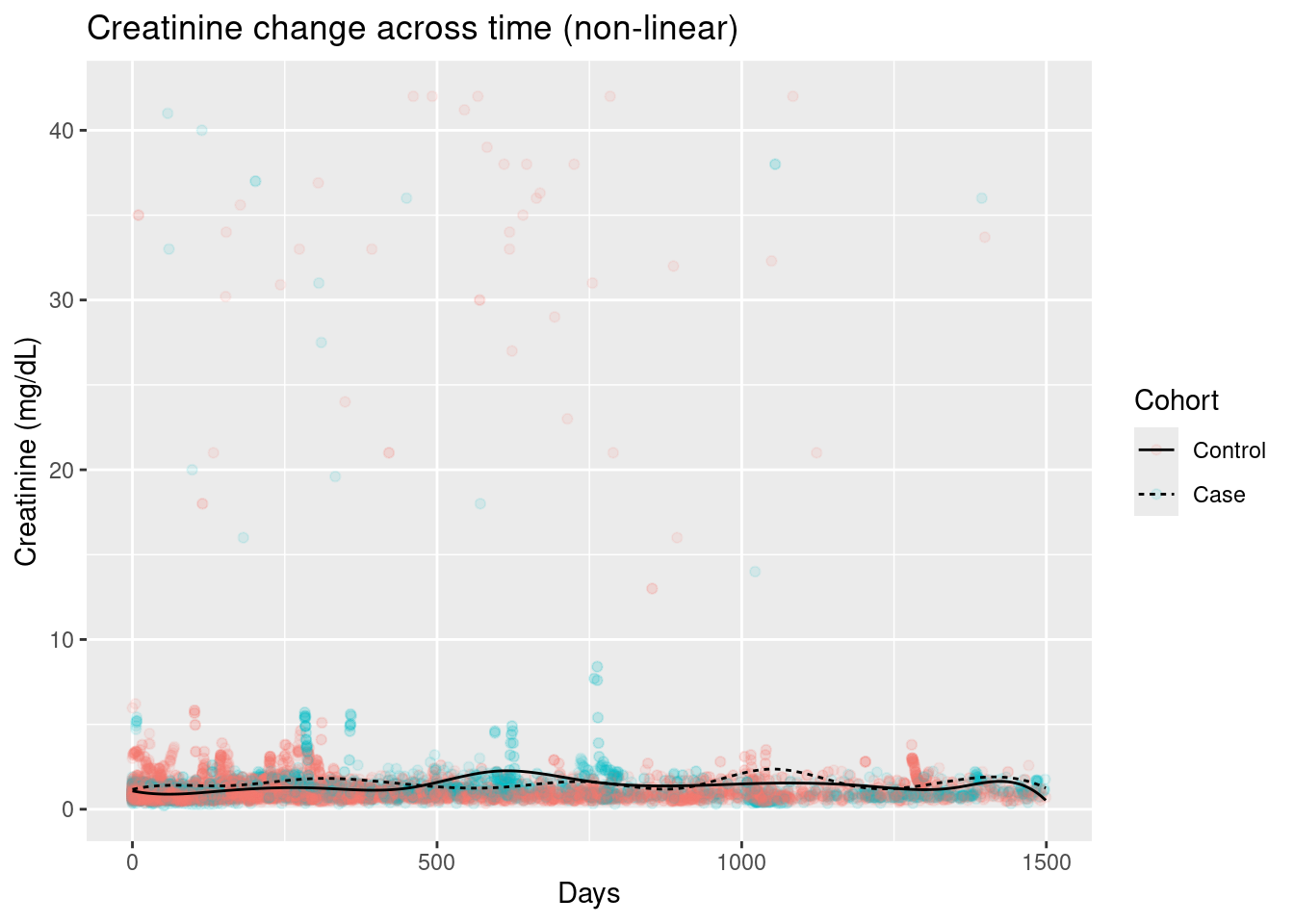

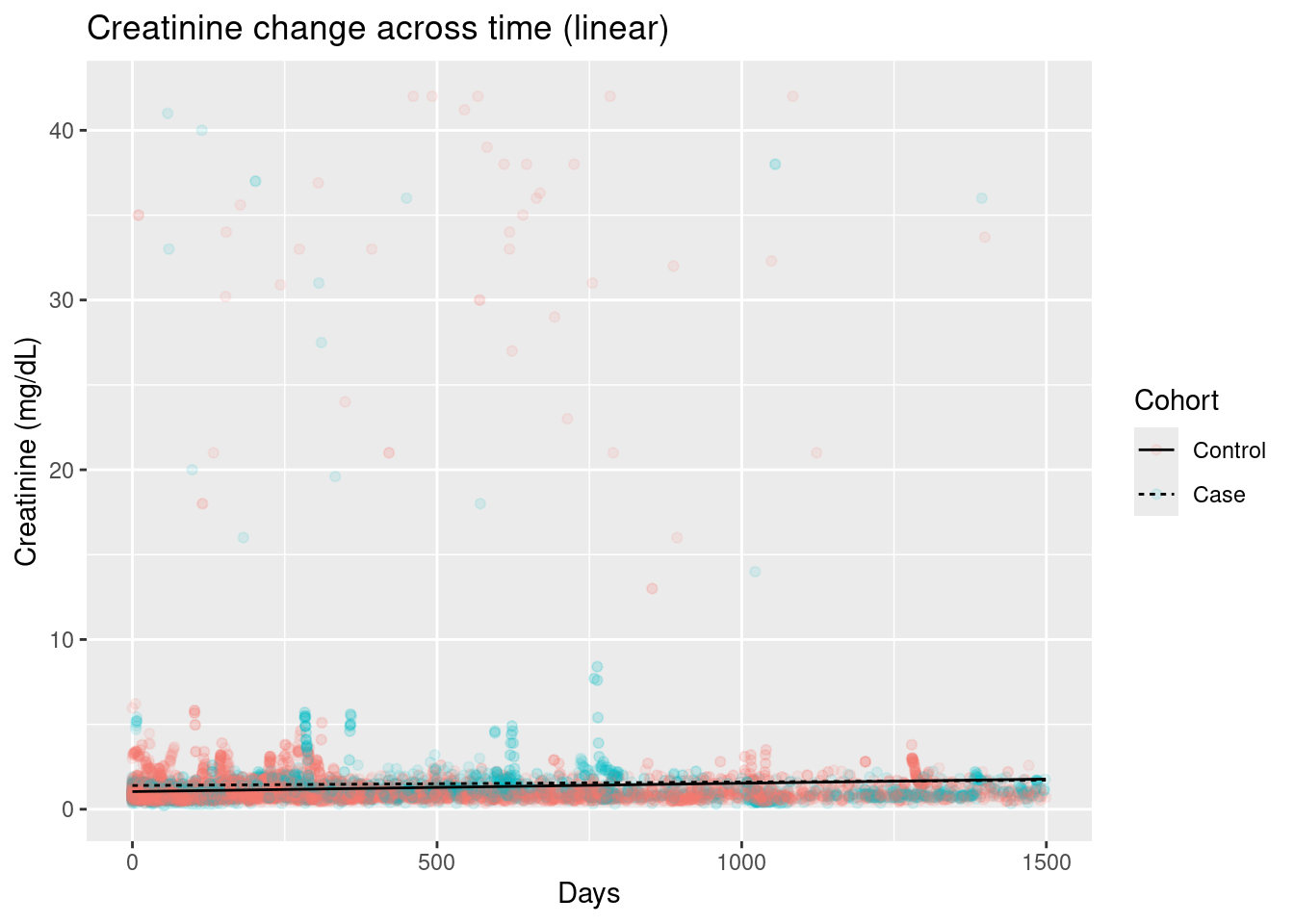
**

Changes in serum creatinine concentrations (mg/dL) over time in diabetic patients treated with anthracyclines with (dotted line) or without (solid line) concomitant glucagon-like peptide-1 (GLP-1) receptor agonist therapy.

**Figure S4: HbA1c Level Changes Across Time Including Patients Where Cancer Stage is Unknown**

**
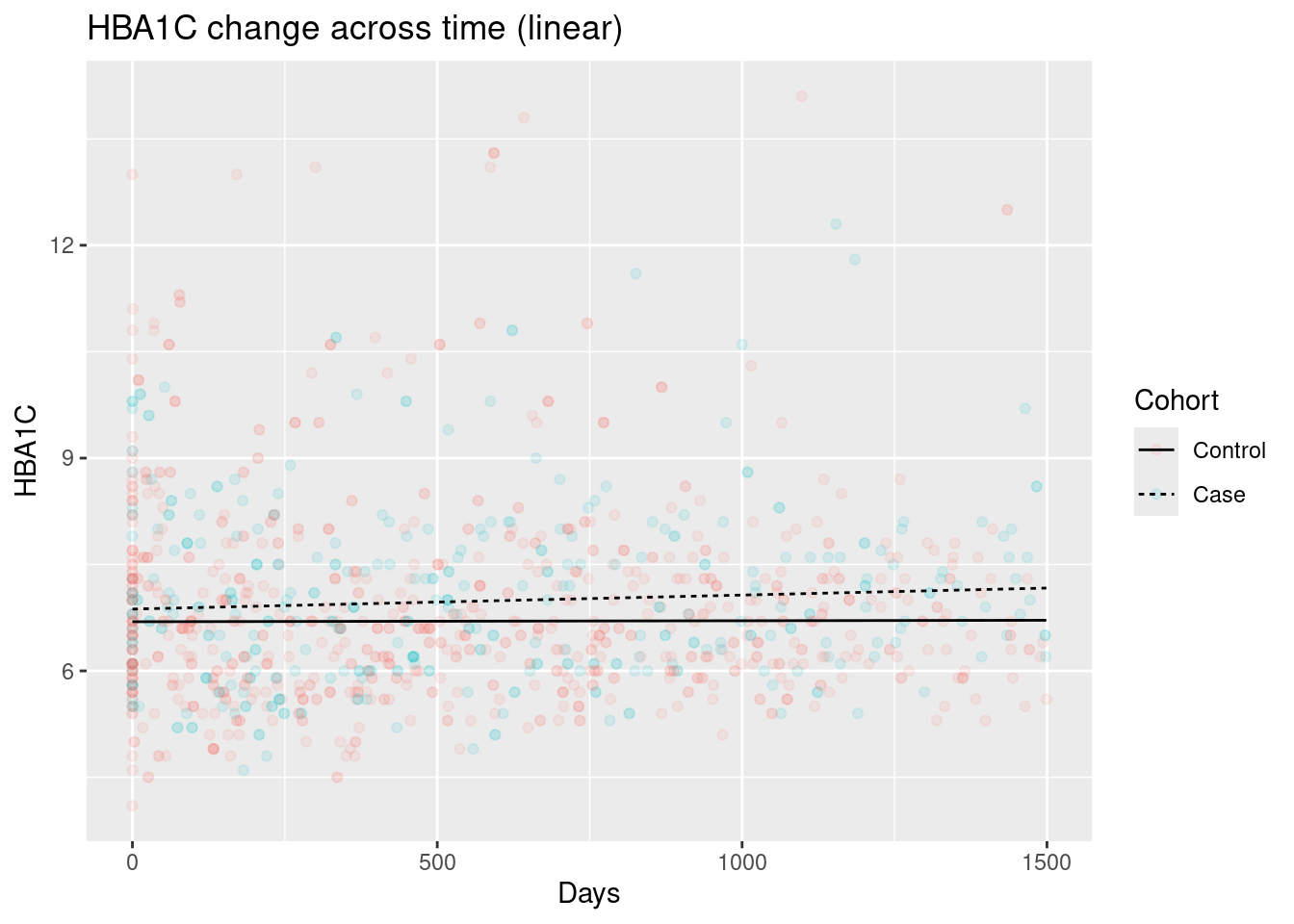
**

**
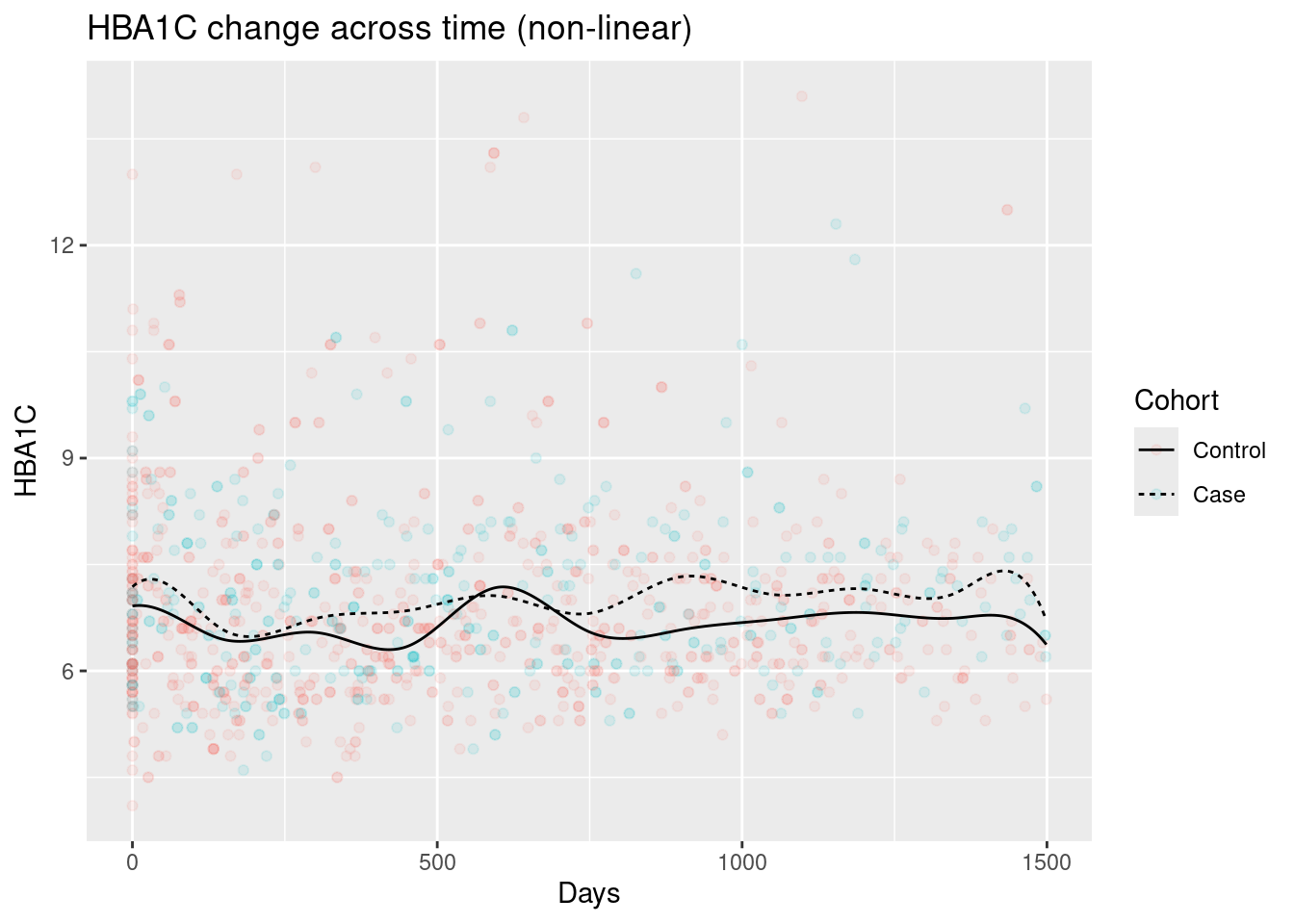
**

Changes in HbA1c level (%) over time in diabetic patients treated with anthracyclines with (dotted line) or without (solid line) concomitant glucagon-like peptide-1 (GLP-1) receptor agonist therapy.

**Figure S5: Cumulative Incidence of 5-Year Mortality Limited to Patients on GLP-1 for at Least 3 Months**


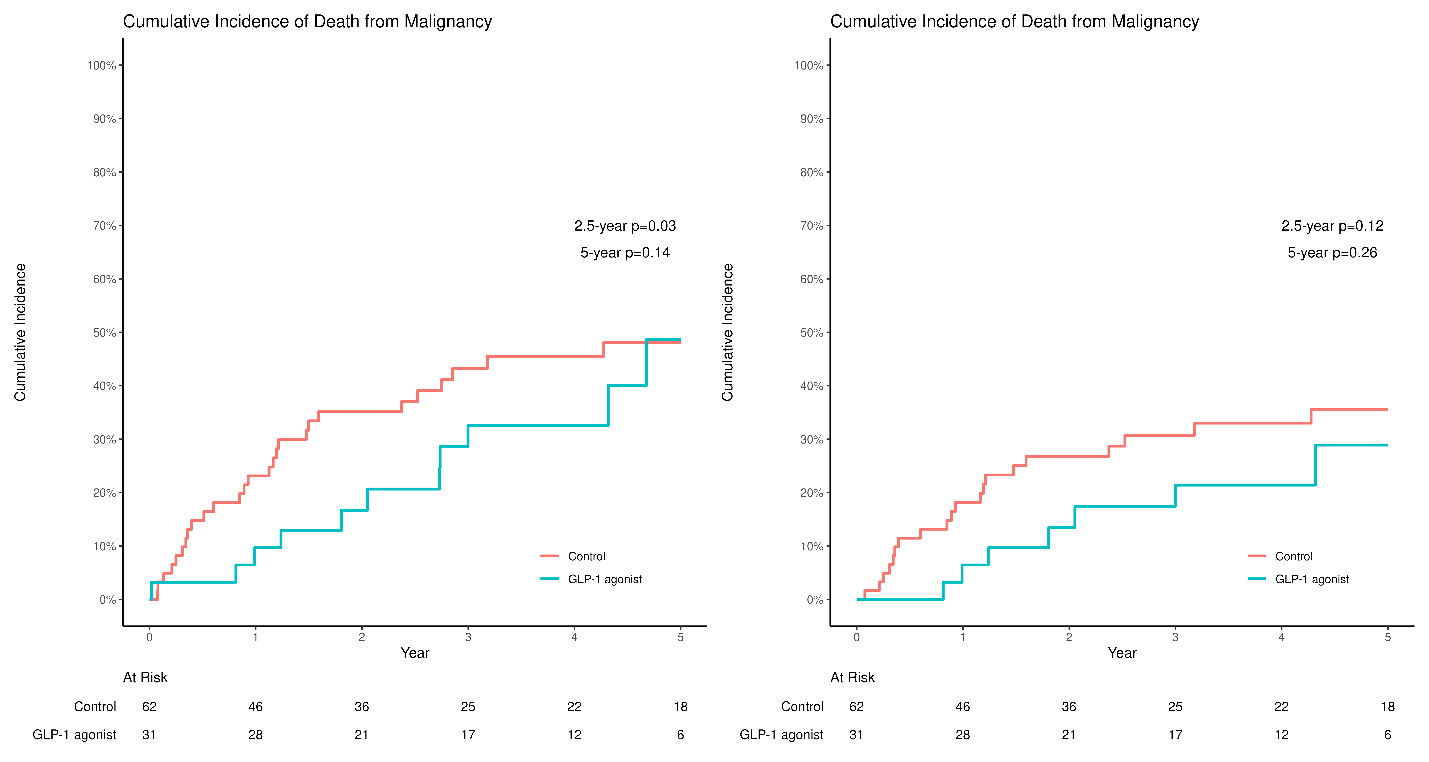


Cumulative incidence curves of 5-year all-cause mortality (left) and death due to malignancy (right) in diabetic patients treated with anthracyclines was compared for those concurrently taking (blue) or not taking (red) a glucagon-like peptide-1 (GLP-1) receptor agonist.

**Table S1. Matched patient baseline characteristics (full cohort including patients with unknown cancer stage)**

|  | GLP-1 RAs  (n=56) | Controls  (n=224) | P |
| --- | --- | --- | --- |
| Age (mean, SD) | 62.8 (10.2) | 65.2 (11.7) | 0.19 |
| Sex, Female (n, %) | 36 (64.3) | 72 (64.3) | 1.00 |
| Malignancy (n, %) |  | | |
| Lymphoma | 13 (23.2) | 36 (32.1) | 0.82 |
| Leukemia | 8 (14.3) | 8 (14.3) |  |
| Breast | 14 (25.0) | 14 (25.0) |  |
| Sarcoma | 1 (1.8) | 1 (1.8) |  |
| Other/Unspecified | 20 (35.7) | 20 (33.9) |  |
| Baseline CVD (n, %) | 26 (46.4) | 89 (39.7) | 0.34 |
| CAD | 12 (21.4) | 48 (21.4) | 1.00 |
| CHF | 0 (0.0) | 0 (0.0) | -- |
| AF | 3 (5.4) | 24 (10.7) | 0.24 |
| Valvular Disease | 17 (30.4) | 54 (24.1) | 0.34 |
| Other Comorbidities (n, %) |  | | |
| HTN | 43 (76.8) | 178 (79.5) | 0.65 |
| HLD | 40 (71.4) | 156 (69.6) | 0.78 |
| Tobacco Use | 23 (41.1) | 118 (52.7) | 0.13 |
| Obesity | 48 (85.7) | 189 (84.4) | 0.78 |
| CKD | 14 (25.0) | 45 (20.1) | 0.40 |
| Concurrent Anti-Glycemics (n, %) |  | | |
| Metformin | 33 (58.9) | 139 (62.1) | 0.60 |
| Sulfonylureas | 18 (32.1) | 41 (18.3) | **0.03** |
| Insulin | 32 (57.1) | 102 (45.5) | 0.11 |
| SGLT2i | 7 (12.5) | 19 (8.5) | 0.26 |
| Concurrent CVD Medications (n, %) |  | | |
| BB | 18 (32.1) | 78 (34.8) | 0.70 |
| ACEi/ARB | 36 (64.3) | 143 (63.8) | 0.64 |
| ARNi | 0 (0.0) | 0 (0) | - |
| Mineralocorticoid | 2 (3.6) | 11 (4.9) | 0.67 |
| Diuretics | 10 (17.9) | 50 (22.3) | 0.45 |
| CCB | 16 (28.6) | 58 (25.9) | 0.68 |
| Statin | 39 (69.6) | 155 (69.2) | 0.93 |
| GLP-1 Agonists |  |  |  |
| Exenatide | 6 (10.7) | - | - |
| Lixisenatide | 1 (1.8) | - | - |
| Oral Semaglutide | 1 (1.8) | - | - |
| Dulaglutide | 13 (23.2) | - | - |
| Exenatide ER | 6 (10.7) | - | - |
| Liraglutide | 18 (32.1) | - | - |
| Semaglutide | 11 (19.6) | - | - |

**Table S2. Risk factor analysis of all-cause mortality (full cohort including patients with unknown cancer stage)**

|  |  | Univariate |  |  | Multivariable |  |
| --- | --- | --- | --- | --- | --- | --- |
|  | HR | CI | P | HR | CI | P |
| Baseline CVD^b^ | 1.87 | 1.30 – 2.70 | **<0.001** |  |  |  |
| CAD | 2.07 | 1.48 – 2.89 | **<0.001** | 1.68 | 0.10 – 2.56 | **0.002** |
| AF | 1.86 | 1.09 – 3.16 | **0.02** | 1.03 | 0.58 – 1.82 | 0.92 |
| Valvular Disease | 1.48 | 1.04 – 2.11 | **0.03** | 1.04 | 0.68 – 1.60 | 0.84 |
| Other Comorbidities |  |  |  |  |  |  |
| HTN | 1.78 | 1.07 – 2.96 | **0.03** | 1.14 | 0.65 – 2.00 | 0.67 |
| HLD | 1.59 | 0.99 – 2.54 | 0.05 | 1.32 | 0.83 – 2.09 | 0.33 |
| Tobacco Use | 1.13 | 0.78 – 1.64 | 0.52 | - | - | - |
| CKD | 1.83 | 1.30 – 2.59 | **<0.001** | 1.54 | 1.00 – 2.41 | **0.049** |
| Anti-Glycemics |  |  |  |  |  |  |
| Sulfonylureas | 0.94 | 0.63 – 1.40 | 0.75 | - | - | - |
| Insulin | 2.00 | 1.35 – 2.95 | **<0.001** | 1.84 | 1.25 – 2.71 | **0.003** |
| GLP-1 RAs | 0.54 | 0.31 – 0.92 | **0.03** | 0.47 | 0.28 – 0.82 | **0.007** |
| Concurrent CVD Medications^b^ |  |  |  |  |  |  |
| BB | 1.13 | 0.78 – 1.66 | 0.50 | - | - | - |
| Mineralocorticoid | 1.17 | 0.54 – 2.54 | 0.69 | - | - | - |
| Diuretics | 1.76 | 1.25 – 2.47 | **0.001** | 1.33 | 0.86 – 2.05 | 0.13 |
| CCB | 1.29 | 0.88 – 1.89 | 0.19 | - | - | - |

^a^Variables with p<0.10 in univariate regression were included in the multivariable model.

^b^CHF was not run due to having no patients in the sample. ARNi was not run due to having only 1 patient in the sample.

**Table S3. Causes of death (full cohort including patients with unknown cancer stage)**

^a^Includes hospice-qualifying diagnoses and complications related to malignancy

^b^Includes other organ failure such as acute kidney injury or respiratory failure

|  | GLP-1 RAs  (n=56) | Control  (n=224) |
| --- | --- | --- |
| Cardiovascular | 0 (0.0) | 5 (2.2) |
| Infectious/Septic | 1 (1.8) | 8 (3.6) |
| Malignancy^a^ | 10 (17.9) | 72 (32.1) |
| Other^b^ | 2 (3.6) | 5 (2.2) |
| Unknown | 5 (8.9) | 23 (10.3) |

**Table S4. Cardiovascular disease hospitalizations (full cohort including patients with unknown cancer stage)**

|  | GLP-1 RAs  (n=56) | Control  (n=224) |
| --- | --- | --- |
| CVD Hospitalizations (n, %)^b^ | 13 (23.2) | 56 (25.0) |
| Atrial Fibrillation | 2 (3.6) | 20 (8.9) |
| Acute Heart Failure | 5 (8.9) | 10 (4.5) |
| Coronary Artery Disease | 2 (3.6) | 23 (10.3) |
| Valvular Disease | 4 (7.1) | 3 (1.3) |
| Composite Cumulative Risk (%)^a^ | 28.0 | 29.1 |

^a^Composite Cumulative Risk Gray’s test p-value = 0.72

^b^Atrial fibrillation included both paroxysmal and permanent atrial fibrillation. Acute heart failure included both systolic and diastolic heart failure. Valvular disease included rheumatic, non-rheumatic, native valve, and prosthetic valve disease.

**Table S5. Matched patient baseline characteristics (patients on GLP-1 RA for at least 3 months)**

|  | GLP-1 RAs  (n=31) | Controls  (n=62) | P |
| --- | --- | --- | --- |
| Age (mean, SD) | 64.1 (9.9) | 65.0 (9.0) | 0.67 |
| Sex, Female (n, %) | 22 (71.0) | 47 (75.8) | 0.63 |
| Malignancy (n, %) |  | | |
| Lymphoma | 9 (29.0) | 22 (35.5) | 0.06 |
| Leukemia | 1 (3.2) | 3 (4.8) |  |
| Breast | 12 (38.7) | 21 (33.9) |  |
| Sarcoma | 7 (22.6) | 1 (1.6) |  |
| Other/Unspecified | 2 (6.5) | 15 (24.2) |  |
| Baseline CVD (n, %) | 16 (51.6) | 26 (41.9) | 0.41 |
| CAD | 6 (19.4) | 15 (24.2) | 0.62 |
| CHF | 0 (0.0) | 0 (0.0) | -- |
| AF | 2 (6.5) | 6 (9.7) | 0.62 |
| Valvular Disease | 11 (35.5) | 18 (29.0) | 0.51 |
| Other Comorbidities (n, %) |  | | |
| HTN | 25 (80.6) | 45 (72.6) | 0.38 |
| HLD | 25 (80.6) | 45 (72.6) | 0.43 |
| Tobacco Use | 15 (48.4) | 36 (58.1) | 0.41 |
| Obesity | 27 (87.1) | 55 (88.7) | 0.81 |
| CKD | 10 (32.3) | 13 (21.0) | 0.18 |
| Concurrent Anti-Glycemics (n, %) |  | | |
| Metformin | 14 (45.2) | 38 (61.3) | 0.53 |
| Sulfonylureas | 8 (25.8) | 13 (21.0) | 0.62 |
| Insulin | 20 (64.5) | 27 (43.5) | 0.06 |
| SGLT2i | 4 (12.9) | 6 (9.7) | 0.64 |
| Concurrent CVD Medications (n, %) |  | | |
| BB | 11 (35.5) | 23 (37.1) | 0.86 |
| ACEi/ARB | 18 (58.1) | 35 (56.5) | 0.86 |
| ARNi | 0 (0) | 0 (0) | - |
| Mineralocorticoid | 0 (0) | 3 (4.8) | 0.99 |
| Diuretics | 7 (22.6) | 12 (19.4) | 0.72 |
| CCB | 10 (32.3) | 17 (27.4) | 0.62 |
| Statin | 22 (71.0) | 46 (74.2) | 0.75 |
| GLP-1 Agonists |  |  |  |
| Exenatide | 3 (9.7) | - | - |
| Lixisenatide | 1 (3.2) | - | - |
| Oral Semaglutide | 0 (0) | - | - |
| Dulaglutide | 8 (25.8) | - | - |
| Exenatide ER | 3 (9.7) | - | - |
| Liraglutide | 12 (38.7) | - | - |
| Semaglutide | 4 (12.9) | - | - |

**Table S6. Risk factor analysis of all-cause mortality (patients on GLP-1 RA for at least 3 months)**

|  |  | Univariate |  |  | Multivariable |  |
| --- | --- | --- | --- | --- | --- | --- |
|  | HR | CI | P | HR | CI | P |
| Baseline CVD^b^ | 1.86 | 0.82 – 4.24 | 0.14 | - | - | - |
| CAD | 3.11 | 1.45 – 6.68 | **0.004** | 2.53 | 1.09 – 5.90 | **0.03** |
| AF | 2.26 | 0.87 – 5.90 | **0.09** | 1.23 | 0.36 – 4.26 | 0.71 |
| Valvular Disease | 1.63 | 0.70 – 3.78 | 0.26 | - | - | - |
| Other Comorbidities |  |  |  |  |  |  |
| HTN | 1.62 | 0.53 – 4.90 | 0.40 | - | - | - |
| HLD | 3.28 | 0.80 – 13.5 | 0.10 | - | - | - |
| Tobacco Use | 1.13 | 0.50 – 2.60 | 0.77 | - | - | - |
| CKD | 2.33 | 1.08 – 5.03 | **0.01** | 2.07 | 0.89 – 4.80 | 0.09 |
| Anti-Glycemics |  |  |  |  |  |  |
| Sulfonylureas | 1.92 | 0.80 – 4.61 | 0.44 | - | - | - |
| Insulin | 1.19 | 0.63 – 2.24 | 0.59 | - | - | - |
| GLP-1 RAs | 0.46 | 0.21 – 1.00 | **0.05** | 0.47 | 0.18 – 1.20 | 0.08 |
| Concurrent CVD Medications^b^ |  |  |  |  |  |  |
| BB | 2.22 | 1.06 – 4.68 | **0.04** | 1.17 | 0.47 – 2.94 | 0.75 |
| Mineralocorticoid | 5.21 | 2.19 – 12.4 | **<0.001** | 3.29 | 0.80 – 13.5 | **0.001** |
| Diuretics | 1.66 | 0.87 – 3.16 | 0.13 | - | - | - |
| CC Blocker | 0.95 | 0.37 – 2.47 | 0.92 | - | - | - |

^a^Variables with p<0.10 in univariate regression were included in the multivariable model.

^b^CHF was not run due to having no patients in the sample. ARNi was not run due to having only 1 patient in the sample.

**Table S7. Causes of death (patients on GLP-1 RA for at least 3 months)**

^a^Includes hospice-qualifying diagnoses and complications related to malignancy

^b^Includes other organ failure such as acute kidney injury or respiratory failure

|  | GLP-1 RAs  (n=31) | Control  (n=62) |
| --- | --- | --- |
| Cardiovascular | 0 (0) | 1 (1.6) |
| Infectious/Septic | 0 (0) | 1 (1.6) |
| Malignancy^a^ | 7 (22.6) | 20 (32.3) |
| Other^b^ | 1 (3.2) | 2 (3.2) |
| Unknown | 4 (12.9) | 4 (6.5) |

**Table S8. Cardiovascular Disease Hospitalizations for Patients on GLP-1 for at Least 3 Months**

|  | GLP-1 RAs  (n=31) | Control  (n=62) |
| --- | --- | --- |
| CVD Hospitalizations (n, %)^b^ | 8 (25.8) | 16 (25.8) |
| Atrial Fibrillation | 0 (0) | 5 (8.1) |
| Acute Heart Failure | 4 (12.9) | 4 (6.5) |
| Coronary Artery Disease | 1 (3.2) | 5 (8.1) |
| Valvular Disease | 3 (9.7) | 2 (3.2) |
| 5-year Composite Cumulative Risk (%)^a^ | 31.8 | 28.0 |

^a^Composite Cumulative Risk Gray’s test p-value = 0.82

^b^Atrial fibrillation included both paroxysmal and permanent atrial fibrillation. Acute heart failure included both systolic and diastolic heart failure. Valvular disease included rheumatic, non-rheumatic, native valve, and prosthetic valve disease.
